# Contamination Characteristics of Antibiotic Resistance Genes in Multi-Vector Environment in Typical Regional Fattening House

**DOI:** 10.3390/toxics12120916

**Published:** 2024-12-18

**Authors:** Kai Wang, Dan Shen, Zhendong Guo, Qiuming Zhong, Kai Huang

**Affiliations:** 1 Research Centre for Livestock Environmental Control and Smart Production, College of Animal Science and Technology, Nanjing Agricultural University, Nanjing 210095, China; wk@stu.njau.edu.cn (K.W.); 2022036@fjtcm.edu.cn (Q.Z.); 2017805091@njau.edu.cn (K.H.); 2 Military Veterinary Research Institute, Academy of Military Medical Sciences, Changchun 130117, China; guozhendong111@gmail.com

**Keywords:** antibiotics resistance gene, metagenomics, fattening house, multi-vector

## Abstract

Antibiotic resistance genes (ARGs) are emerging as significant environmental contaminants, posing potential health risks worldwide. Intensive livestock farming, particularly swine production, is a primary contributor to the escalation of ARG pollution. In this study, we employed metagenomic sequencing and quantitative polymerase chain reaction to analyze the composition of microorganisms and ARGs across four vectors in a typical swine fattening facility: dung, soil, airborne particulate matter (PM), and fodder. Surprisingly, soil and PM harbored a higher abundance of microorganisms and ARGs than dung. At the same time, fodder was more likely to carry eukaryotes. Proteobacteria exhibited the highest propensity for carrying ARGs, with proportions 9–20 times greater than other microorganisms. Furthermore, a strong interrelation among various ARGs was observed, suggesting the potential for cooperative transmission mechanisms. These findings underscore the importance of recognizing soil and PM as significant reservoirs of ARGs in swine facilities alongside dung. Consequently, targeted measures should be implemented to mitigate their proliferation, mainly focusing on airborne PM, which can rapidly disseminate via air currents. Proteobacteria, given their remarkable carrying capacity for ARGs with the primary resistance mechanism of efflux, represent a promising avenue for developing novel control strategies against antibiotic resistance.

## 1. Introduction

Industrialized livestock operations generate vast animal waste, including manure, urine, and other organic residues. These waste products accumulate in large quantities and can release pollutants such as nitrogen, phosphorus, pathogens, and antibiotic residues into the surrounding soil, water, and air, bringing a myriad of environmental challenges. China produced 20 million tons of dung in 2017 [1]. Swine dung represents the most significant single dung source, with an estimated 6 million tons annually. It continues to expand [2]. It usually harbors pathogens like *Salmonella*, *Escherichia coli*, and *Enterococcus*, so biosafety is a concern [3]. In addition, some other vectors in swine houses, airborne particulate matter (PM), soil, and fodder, are also of concern for biosafety. Soil from swine farms is also often rich in pathogens due to fecal contamination. According to the report, 2–6% of potentially pathogenic bacteria and as high as 20–50% of allergenic fungi are carried in airborne PM_2_._5_ (PM with aerodynamic diameter ≤2.5 µm) of swine houses [4]. In fodder, paramount attention is directed towards mycotoxin-producing fungi, notably *Aspergillus* and *Fusarium*. The hazards become even more formidable when these potential pathogenic microorganisms harbor antibiotic-resistant genes (ARGs).

The widespread dissemination and accumulation of ARGs in multidrug-resistant pathogens pose a significant challenge to the lifesaving efficacy of antibiotics, thus threatening public health. The surge in the environmental reservoir of ARGs is attributed mainly to the indiscriminate use of antibiotics. China is the largest producer and consumer of antibiotics in the world. More than 30,000 tons of antibiotics were used in livestock farming in 2020, far more than in any other country [5]. In addition to therapeutic purposes, antibiotics are extensively used as feed additives in animal husbandry for growth promotion and disease prevention. The excessive intake of antibiotics leads to an increase in corresponding ARGs in animal dung, which subsequently spread to soil and vegetables treated with dung, downstream rivers, and sediment [2], posing a rapid threat to human health. Most studies on the pollution of ARGs in livestock farms mainly focus on vectors such as dung, soil, and sewage. At the same time, there is a lack of sufficient research on airborne PM. Limited investigation has revealed that high concentrations of ARGs in airborne PM are positively correlated with fecal ARGs in swine farms [6,7], indicating that PM also serves as a significant vector within farming facilities. The PM can remain suspended in the air for extended periods, facilitating the widespread dissemination of ARGs with air movement. In short, ARGs from farming facilities can impact humans through multiple transmission pathways. Recent studies have found a close link between ARGs in farm animals and farmers, confirming the severe threat posed by ARGs generated in livestock farming to human health [8]. ARGs’ rapid and extensive spread is attributed to their complex transmission mechanisms, which can occur through multiple pathways. Direct contact and shared living spaces among animals facilitated the direct transmission of resistant bacteria and their genetic materials. Contaminated dung, airborne PM, soil, fodder, and water serve as reservoirs for antibiotic-resistant bacteria and their genetic elements, enabling indirect transmission across animal populations. The vertical inheritance from parents to offspring contributes to the persistence of ARGs within livestock populations across generations. Additionally, horizontal gene transfer mediated by mobile genetic elements, such as plasmids and transposons, among microorganisms plays a central role in spreading ARGs within animal houses. Investigations indicate that tetracycline and sulfonamide resistance genes in swine dung, often harbored within highly mobile plasmids, exacerbate horizontal transmission within swine populations [9].

The epidemiology and transmission pathways of ARGs are diverse. They can involve human-to-human, animal-to-human, and environmental dissemination routes. The interconnectedness of these reservoirs underscores the need for a holistic approach to address antibiotic resistance. To effectively curb the production of antibiotic-resistant bacteria and ARGs in livestock farming, China has prohibited fodder manufacturers from producing commercial fodder additives containing growth-promoting drugs, marking the advent of an era of antibiotics restriction in animal husbandry [10]. However, based on the experience of the European Union, the ban on antibiotics in fodder may lead to a significant increase in their use at the treatment end for therapeutic purposes, underscoring the continued ecological risks. Currently, research on antibiotic resistance gene contamination in the livestock industry primarily focuses on dung, with limited attention paid to other vectors within the facilities. There is scarce analysis of the attribution relationship between ARGs and antibiotic-resistant bacteria. This study utilized metagenomic sequencing and real-time quantitative polymerase chain reaction (RT-qPCR) to investigate the contamination characteristics of bacterial communities and ARGs in various vectors within fattening facilities, elucidating the attribution relationship between ARGs and microorganisms. This research provides a practical foundation for studying the impact of ARGs originating from swine facilities on the surrounding environment. It lays the theoretical groundwork for mitigating the emission and dissemination of ARGs in the future.

## 2. Materials and Methods

### 2.1. Animal Ethics

All sampling procedures in this experiment have been endorsed by the Animal Ethics Committee of Nanjing Agricultural University (SYXK (su) 2021-0086).

### 2.2. Fattening House Description

**Figure 1:**
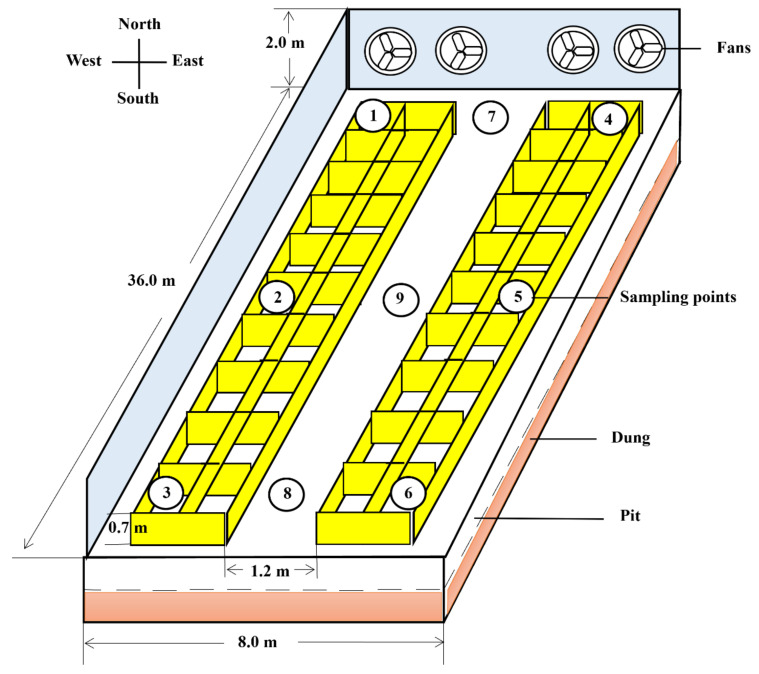
Plan view of the fattening house showing the sampling locations for dung, soil, particulate matter (PM), and fodder. ①, ②, ③, ④, ⑤, ⑥ for dung and fodder; ⑦, ⑧ for soil; ⑨ for PM. The diagram was not drawn to scale.

Considering the distribution of China’s pig farming industry, which exhibits regional variations in productivity levels due to inherent limitations and growth potential [11], Hebei Province stands out as a critical region, playing a pivotal role in the nation’s pig industry. Over the past seven years, it has consistently accounted for approximately 5% of the country’s total pork production (China Statistical Yearbook, 2016–2023). Adopting typical fattening technologies and the representative pigsty planning and allocation strategies employed in this province embody the advancements made in China’s pig farming sector [12]. We randomly selected one of these farms to serve as a case study for our study. This study was conducted in a commercial enclosed fattening house located in Shijiazhuang City, Hebei province, China (east longitude 113°30′ to 115°20′, north latitude 37°27′ to 38°47′). The fattening house is oriented north–south with dimensions of 36 m × 8 m (Figure 1). The house has a tunnel ventilation system consisting of wet curtains and fans installed in the north- and south-end walls, respectively. A warm air stove was placed outside the house, and a windpipe system was used for heating in winter. There is a 1.2 m wide aisle in the middle of the house and 10 pens on the east and west sides, each of which housed 10–12 pigs, a total of about 250 fattening pigs aged from 120 to 160 days old and weighing approximately 80 kg. The feeding cylinder was mechanically operated daily by the farmer for pigs taking pellet feed at 6:00 am and 5:00 pm, and the health of herds was checked simultaneously. Fecal cleaning was performed daily at 3:00 pm.

### 2.3. Sample Collection

The PM samples (TSP, PM with aerodynamic diameter ≤100 µm) were continuously collected on the quartz filter membranes for 3 days by an air particle sampler (HH02-LS120, Huarui Hean, Beijing, China) with a high flow rate of 1000 L/min at a height of 1.0 m in the middle of the fattening house (Figure 1). The filter membranes with the PM were cut into pieces and used directly to extract DNA like any other samples. Dung and fodder samples were collected separately from pens in the front, middle, and rear parts of both sides of the fattening house. Dung was collected fresh, and only the middle portion was preserved. Soil samples were collected from the front and rear of the aisle. Vector samples were collected from the multiple positions mentioned above 3 times per month and thoroughly mixed as one replicate. A total of 3 replicates for each vector were obtained in September, October, and November 2018, respectively. Fresh samples were collected into sterile tubes, snap-frozen by liquid nitrogen, and kept at −80 °C for metagenome analysis.

### 2.4. Metagenome Analysis

DNA extraction: The total genomic DNA was extracted from 0.3 g of each sample weighed using a soil genomic DNA extraction kit (Axygen, Union City, CA, USA) following the manufacturer’s instruction.

Library construction: A total amount of 1 μg DNA per sample with qualified purity and concentration was fragmented by sonication to a size of 350 bp for Illumina sequencing with further PCR amplification. The PCR products were purified with the AMPure XP system (Beckman, CA, USA), and libraries were constructed (Agilent 2100 Bioanalyzer, Santa Clara, CA, USA).

Sequencing: The library preparations were sequenced on an Illumina NovaSeq platform and generated paired-end reads.

Metagenome assembly: The raw data obtained from the sequencing platform were preprocessed using Readfq (V8, https://github.com/cjfields/readfq, accessed on 10 December 2020) to acquire the Clean Data, which were subsequently assembly analyzed by SOAPdenovo software (V2.04, https://hcc.unl.edu/docs/applications/app_specific/bioinformatics_tools/de_novo_assembly_tools/soapdenovo2/, accessed on 10 December 2020).

Gene prediction and abundance analysis: An ORF prediction for the Scaftigs (≥500 bp) assembled from both single and mixed samples was conducted using MetaGeneMark (V2.10, http://topaz.gatech.edu/GeneMark/, accessed on 10 December 2020). Basic information statistics and Venn diagram analysis of gene numbers were performed based on the abundance of information on each gene in each sample in the obtained gene catalog (Unigenes).

Taxonomy annotation: The Unigenes were aligned with sequences of Bacteria, Fungi, Archaea, and Viruses extracted from the NR database (Version 2018-01-02: http://www.ncbi.nlm.nih.gov/, accessed on 10 December 2020) of NCBI using DIAMOND software (v0.99.110, https://github.com/bbuchfink/diamond/, accessed on 10 December 2020). The PCA (R package, Version 2.15.3) and PCoA (R package, Version 2.15.3) decrease-dimension analysis were exhibited based on the abundance tables of each taxonomic hierarchy. The Metastats and LEfSe (LEfSe software, the default LDA score was 3) methods were used to find different species between groups. Functional annotations were obtained by aligning Unigenes with the Kyoto Encyclopedia of Genes and Genomes (KEGG) database (Version 2018-01-01, http://www.kegg.jp/kegg/, accessed on 10 December 2020) using DIAMOND software (v0.99.110, https://github.com/bbuchfink/diamond/, accessed on 10 December 2020).

Resistance gene annotation: Unigenes were compared with the CARD database (https://card.mcmaster.ca/, accessed on 10 December 2020) using Resistance Gene Identifier (RGI) software (version 4.0.3, McMaster University, Hamilton, ON, Canada, 2018) to calculate the relative abundance of ARO, based on which the abundance histogram, overview graph, clustering heatmap, and identification circle diagram were obtained.

### 2.5. PCR Assay

Real-time quantitative PCR was employed to quantify the abundances of 8 representative ARGs, including *tet32*, *tet40*, *tetQ*, *tetL*, *aph3′-ia*, *aph3′-iiia*, *floR,* and *optrA* genes in fodder, dung, PM, and soil samples; the primer sequences are listed in Table S1. Sterile water was used as the negative control for each qPCR run. The PCR reactions were performed in 25 μL of reagent containing 1 μL template DNA, 1 μL of each primer, 12.5 μL Premix Taq™ (TaKaRa, Kyoto, Japan), and 9.5 μL RNase-free Water. Annealing temperatures were different for different ARGs, as shown in Table S1. After amplification, the total amount of PCR products (50 μL) was verified for purity and primer specificity by 1.5% (*w*/*v*) agarose gel electrophoresis (Figure S1) and purified using an Eastep^TM^ Gel and PCR Clean-up kit (Promega, Wisconsin, USA) according to the manufacturer’s instruction. The purified target ARG PCR products were ligated into the pMD18-T vector (TaKaRa, Kyoto, Japan) and cloned into 100 μL of Trans5α competent cells (TaKaRa, Kyoto, Japan). Following screening, the recombinant bacterial clones were sent to Kumei Co., Ltd. (Changchun, China) for sequencing to confirm the gene insertions. Plasmids containing the target genes were extracted and used to construct standard curves for qPCR quantification. Each gene copy number was quantified using the Applied Biosystems QuantStudio™^1^ qPCR system (ABI, Los Angeles, CA, USA). The qPCR (10 μL) system contained 5 μL of 2× Power SYBR Green PCR Master Mix (ABI, CA, USA), 0.2 μL of each primer, 1 μL of DNA template, and 3.6 μL of ddH_2_O. The thermal cycling conditions were 98 °C for 10 min, followed by 40 cycles of 98 °C for 30 s, 60 °C for 30 s, and 72 °C for 30 s. Each sample was analyzed in triplicate. The plasmid DNA containing the target genes was serially diluted 10-fold to generate the standard curves, all of which had R^2^ values greater than 0.995. A Ct value of 31 was set as the detection limit, and targets detected in at least two out of three replicates with a deviation of less than 20% were considered positive. Gene copy numbers of plasmids were calculated according to the following formula:

Nc (copies/mL) = (N_A_ × C × 10^−9^)/(L × M_bp_)

Among them, Nc = plasmid gene copy number; N_A_ = Avogadro’s constant, which is 6.02 × 10^23^ mol^−1^; C = recombinant plasmid concentration; L = the recombinant plasmid fragment size, which is equal to the sum of the vector fragment size and the inserted DNA fragment size; M_bp_ = the average molecular weight of each pair of bases, which is 660. The linear regression equation for calculating the copy number of the target ARGs is shown in Table S2.

### 2.6. Statistical Analysis

Data were statistically analyzed, and graphs other than omics were plotted using SPSS version 25 and GraphPad Prism version 8, respectively. Statistical differences were calculated by one-way ANOVA followed by Tukey’s multiple comparison [13]. Values were expressed by mean ± standard error of the mean (SEM), and *p* < 0.05 was defined as a statistically significant difference.

## 3. Results

### 3.1. Microbial Gene Number and Microbial Components in Different Vectors

**Figure 2:**
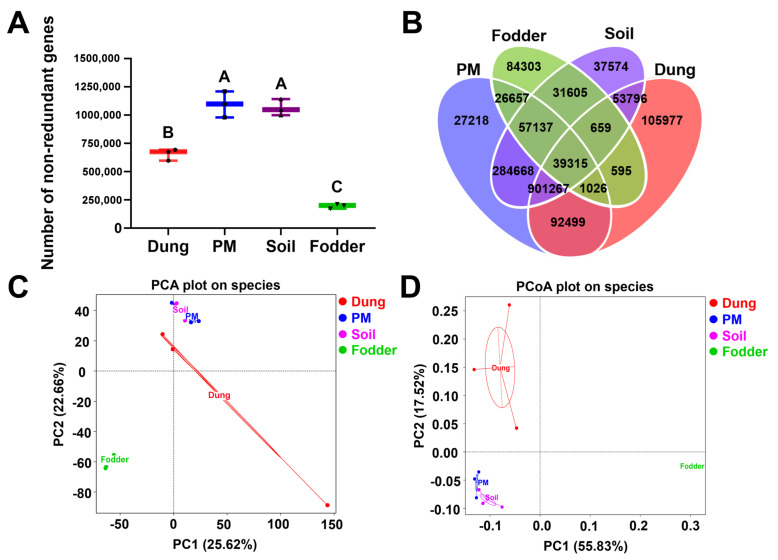
The number of microbial genes and differences of microbial components in different vectors in the fattening swine house. (**A**) Number of non-redundant genes in dung, PM, soil, and fodder in fattening swine house. Different capital letters represent significant differences between vectors (*p* < 0.01); the same letters show no significant differences (*p* > 0.05). (**B**) A Venn diagram showing the number of standard and unique genes in dung, PM, soil, and fodder in a fattening swine house. (**C**) Principal component analysis (PCA) based on a linear model was plotted to display microbial composition differences among species-level samples. (**D**) Principal coordinate analysis (PCoA) based on the Bray–Curtis distance matrix of species. n = 3 per vector.

As shown in Figure 2A, the total number of microbial genes was highest in PM and soil in the fattening swine house, which was significantly higher than that in dung and fodder, and was lowest in fodder, which was significantly lower than that in the other three vectors (*p* < 0.01). The number of standard and unique genes in four vectors is shown in Figure 2B. The highest number of unique genes was observed in dung, up to 105,977. A total of 27,218 unique microbial genes were detected in PM, which was the lowest. The number of mutual genes in PM, soil, and dung was as high as 901,267.

Microbial diversity analysis revealed that the main components of microorganisms in PM and soil were similar. In contrast, the principal components in dung and fodder were significantly different from those in the other three vectors based on the PCA (Figure 2C) and PCoA (Figure 2D) plots on the species level.

### 3.2. Dominant Microbiota in Different Vectors

**Figure 3:**
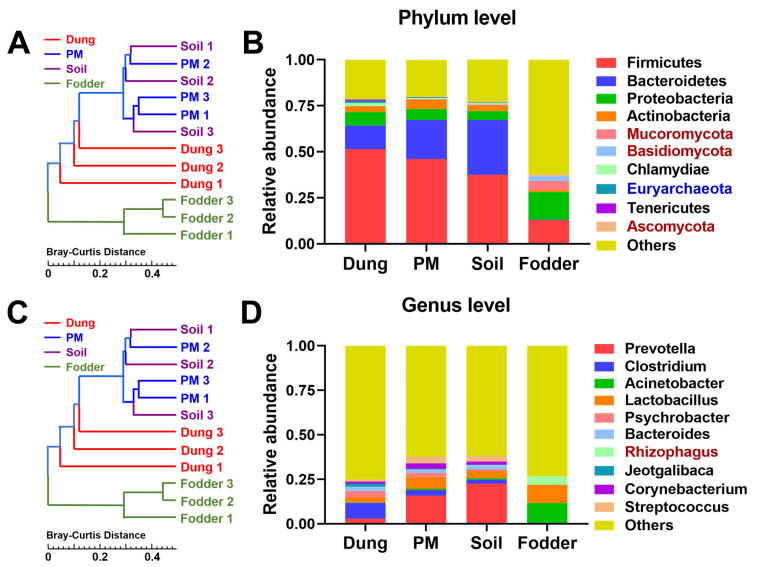
Clustering and relative abundance of microbial taxa in dung, PM, soil, and fodder in the fattening swine house. (**A**) Hierarchical clustering tree of microbial taxa at the phylum level. (**B**) Relative abundance of the top 10 most dominant microbial taxa at the phylum level. (**C**) Hierarchical clustering tree of microbial taxa at the genus level. (**D**) Relative abundance of the top 10 most dominant microbial taxa at genus level. n = 3 per vector. Microbes with black, red, and blue fonts indicate that they belong to Bacteria, Eukaryotes, and Archaea kingdoms.

The hierarchical clustering results were similar to those of microbial diversity analysis. On both the phylum (Figure 3A) and genus levels (Figure 3C), the microbial distribution of dung and fodder groups was completely separated from the other three groups. At the same time, they were separated from each other into PM and soil groups.

Specifically, Figure 3B shows that Firmicutes and Bacteroidetes were the dominant phyla in dung, PM, and soil. On the contrary, the predominant phyla in fodder were Firmicutes and Proteobacteria. The key genera contributing to the differences in the principal components of microbiota in each vector are shown in Figure 3D. All of the second dominant genera were *Lactobacillus*. In contrast, the first dominant genera were different from one another among the four vectors. *Clostridium* and *Acinetobacter* were the first dominant genus in dung and fodder, respectively. Consistent with the results of principal component analysis, the dominant genera in the PM and soil were the same, *Prevotella*.

### 3.3. Differential Microbiota in Different Vectors

**Figure 4:**
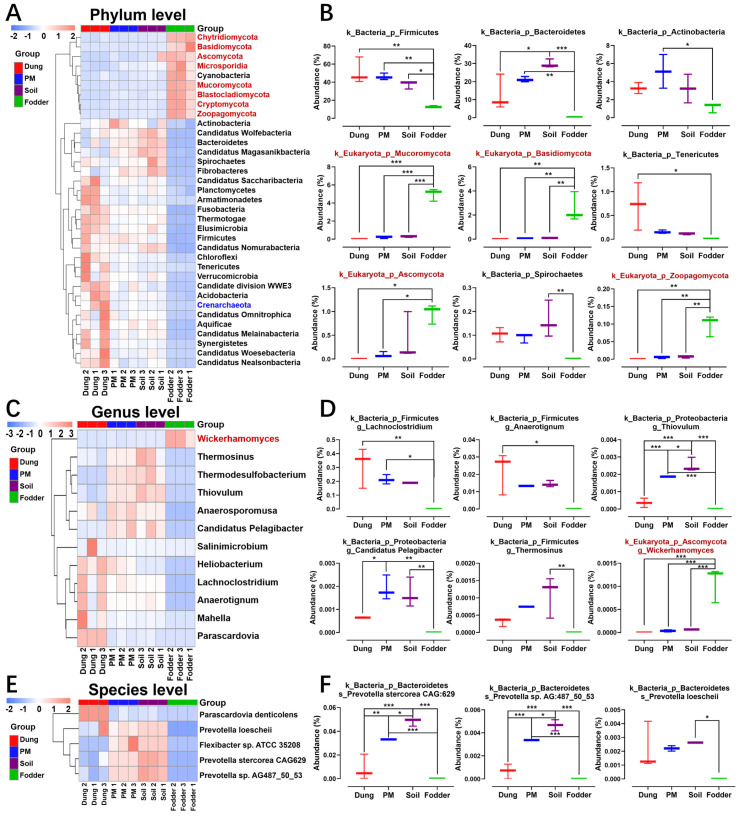
Clustering and abundance comparison of differential microbes at different levels in dung, PM, soil, and fodder in the fattening swine house based on the Metastats analysis method. (**A**) Clustering heatmap of differential microbial abundance at the phylum level. (**B**) Comparison of differential microbial abundance at the phylum level (the top 9 most dominant differential phyla). (**C**) Clustering heatmap of differential microbial abundance at the genus level. (**D**) Comparison of differential microbial abundance at the genus level (the top 6 most dominant differential genera). (**E**) Clustering heatmap of differential microbial abundance at the species level. (**F**) Comparison of differential microbial abundance at the species level (the top 3 most dominant differential species). * *p* < 0.05, ** *p* < 0.01, *** *p* < 0.001. n = 3 per vector. Microbes with black, red, and blue fonts indicate that they belong to the Bacteria, Eukaryotas, and Archaea kingdoms.

There were thirty-five, twelve, and five differential microorganisms at the phylum (Figure 4A), genus (Figure 4C) and species (Figure 4E) levels, respectively. The clustering heatmap of the differential microbiota showed that the microorganisms in dung, PM, and soil were mainly bacteria. At the same time, those in fodder were mainly eukaryotes (Figure 4A,C). The phyla, genera, and species of bacteria with high abundance in dung significantly differed from those in PM and soil.

The top nine most dominant differential phyla are shown in Figure 4B, including five bacteria of Firmicutes, Bacteroidetes, Actinobacteria, Tenericutes, and four eukaryotes of Mucoromycota, Basidiomycota, Ascomycota, Zoopagomycota. Of the six most dominant microbial genera, five belong to bacteria, and one belongs to eukaryotes (Figure 4D). At the species level, all three differential species with the highest abundance belong to *Prevotella*, in which the abundance of *Prevotella stercorea CAG: 629* and *Prevotella* sp. *AG: 487_50_53* in PM and soil is significantly higher than in dung and fodder (Figure 4F, *p* < 0.05).

### 3.4. Featured Microbial Taxa in Different Vectors

**Figure 5:**
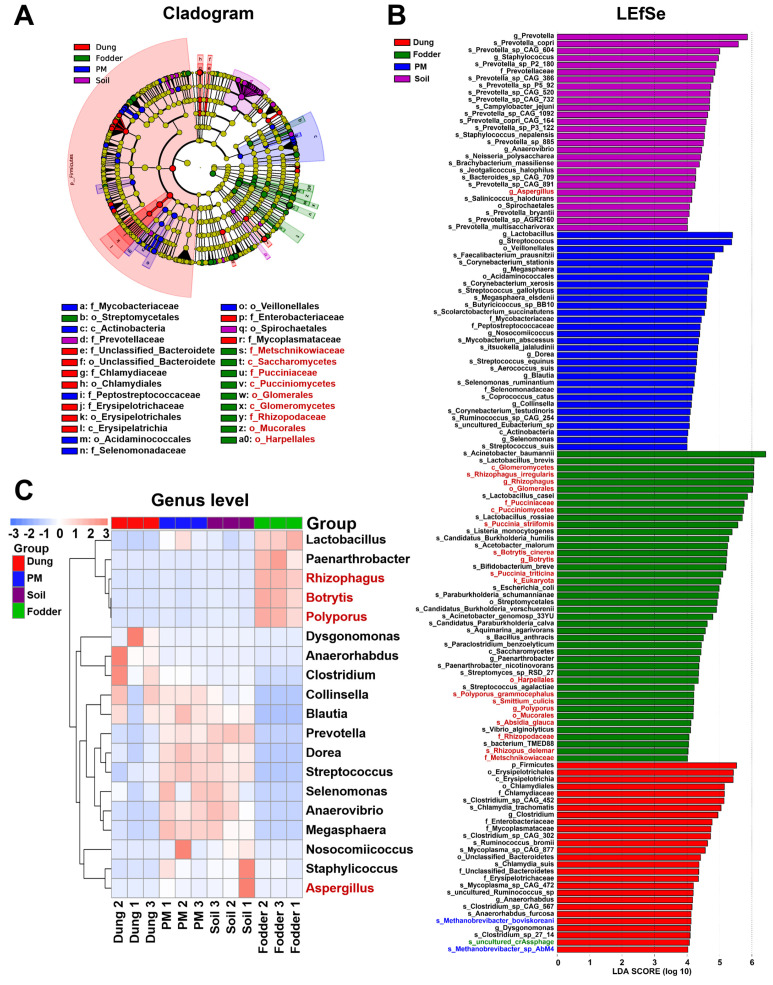
Screening of microbial biomarkers with significant differences among different vectors in the fattening swine house based on the linear discriminant analysis (LDA) effect size (LEfSe) analysis. (**A**) The cladogram shows the significantly enriched microbial taxa (from class to family level). (**B**) The bar chart shows the LDA score of microbial taxa (from kingdom to species level) in dung, PM, soil, and fodder. (**C**) Clustering heatmap of featured microbial abundance at the genus level. Significant differences were defined as *p* < 0.05 and LDA score > 4.0. n = 3 per vector. Microbes with black, red, blue, and green fonts indicate that they belong to the kingdoms of Bacteria, Eukaryotas, Archaea, and Viruses, respectively.

The cladogram indicated that 27 featured microbes from the class to the family level were detected in four vectors (Figure 5A). All nine eukaryotes were discovered in fodder. The linear discriminant analysis (LDA) effect size (LEfSe) method was utilized to estimate the contribution of the abundance of each microbial component in more detail from the kingdom to the species level on the difference effect (Figure 5B). A total of 28 different features in soil were revealed, including one eukaryote, *Aspergillus*, and twenty-seven bacteria, with seventeen genera or species of *Prevotella*. There are 31 different features in PM, all of which are bacteria, of which *Lactobacillus* contributed the most. Unsurprisingly, of the 44 features in the fodder, 20 were eukaryotes. More types of featured microbial taxa were detected in dung than in other vectors, with two archaeal species and one viral species in addition to the twenty-four bacteria.

All nine featured genera were clustered in the heatmap (Figure 5C). The highly abundant genera in PM and soil were similar. The featured genera in fodder, *Lactobacillus*, *Paenarthrobacter*, *Rhizophagus*, *Botrytis*, and *Polyporus*, were mainly fungi, clearly distinguished from the other three vectors.

### 3.5. Microbial Functional Prediction in Different Vectors

**Figure 6:**
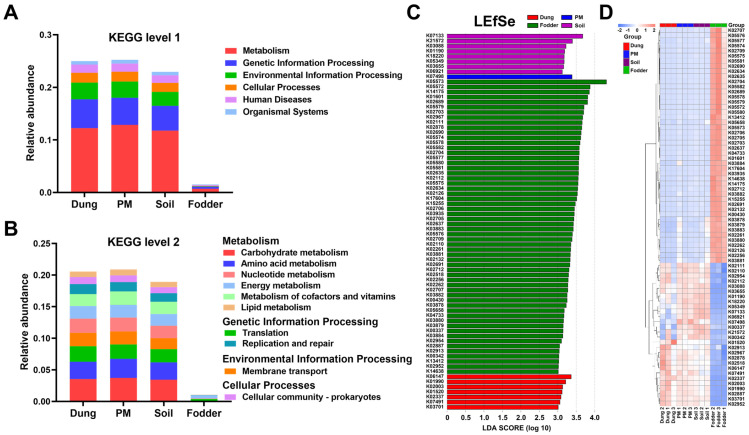
Microbial functional relative abundance and screening and clustering of differential func-tions in dung, PM, soil, and fodder in the fattening swine house. (**A**) Relative abundance of microbial functional annotations at KEGG level 1. (**B**) Relative abundance of microbial functional annotations at KEGG level 2. (**C**) The bar chart shows the LDA score of significantly differential functions at the KO (KEGG orthology) level. Significant differences were defined as *p* < 0.05 and LDA score > 3.0. (**D**) Clustering heatmap of differential microbial functions at KO level. n = 3 per vector.

A relative abundance of microbial functional annotations at KEGG levels 1 and 2 is displayed in Figure 6A,B, respectively. The most microbial-abundant function at level 1 was mainly metabolism, followed by genetic information processing in all four vectors. Carbohydrate metabolism was the most abundant function at level 2, followed by amino acid metabolism and translation in dung, PM, and soil. The most abundant functions at level 2 in fodder were energy metabolism and translation, which differed from the other three vectors. The abundance of functional annotations in fodder was visibly lower than the other three vectors, which may be associated with the lowest number of microbial genes.

The LEfSe analysis revealed that eight, fifty-eight, one, and seven featured proteins of homologous genes in soil, fodder, PM, and dung, respectively (Figure 6C). The detailed functions and pathways of 74 featured proteins are listed in Table S3. Of eight featured proteins of soil, two belonged to metabolism function, one belonged to genetic information processing function, one was antimicrobial resistance genes, and four had unknown functions or pathways. Noteworthily, the fodder contained the highest amount of featured protein, more than 7-fold that of the other vectors. Of the 58 featured proteins, 45 belonged to metabolism function. Some of these proteins also have other functions. The only featured protein in the PM has an ambiguous function. Among the seven featured proteins in dung, one had a metabolism function, one had a genetic information processing function, one had two functions, and four had unknown functions. Clustering the abundance of these proteins revealed 46 featured proteins that were very clearly highly abundant only in fodder (Figure 6D). Also, 15 highly abundant featured proteins were discovered in PM and soil, and 13 were found in dung.

### 3.6. ARG Number and ARO Components in Different Vectors

**Figure 7:**
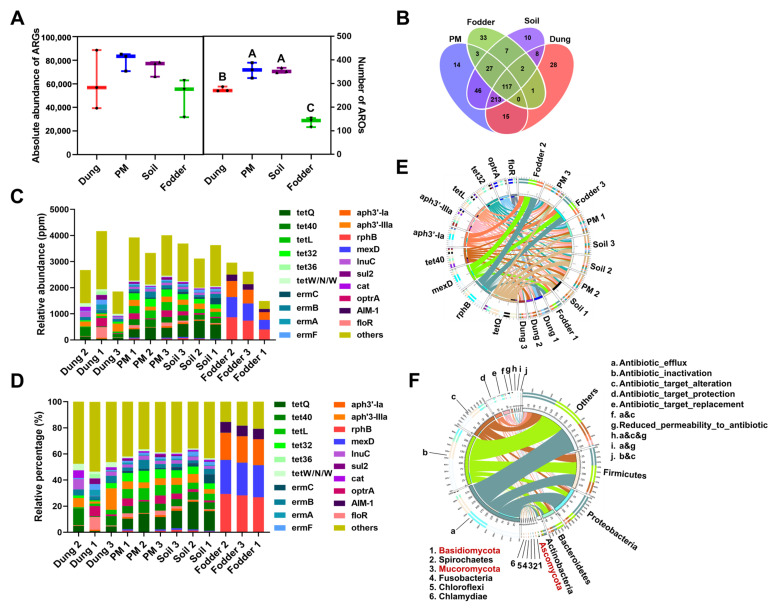
The relative abundance of antibiotic resistance genes (ARGs) in dung, PM, soil, and fodder in the fattening swine house. (**A**) Absolute abundance of ARGs (left) and several antibiotic resistance ontologies (AROs) (right). Different capital letters represent significant differences between vectors (*p* < 0.01); the same letters show no significant differences (*p* > 0.05). (**B**) A Venn diagram showed the common and unique AROs among different vectors. (**C**) The relative abundance of the top 20 most dominant AROs accounted for all genes in each sample (The original relative abundance data were magnified 10^6^ times). (**D**) The top 20 dominant AROs accounted for the relative percentage of all AROs. (**E**) An overview graph was plotted to display each sample’s abundance ratio of the top 10 most dominant AROs. The left inner and outer circles in the diagram represent the summation of the relative abundance and relative percentage content of AROs across various samples, respectively. On the right side, the summation of the relative abundance and relative percentage content of each ARO within a sample is shown. (**F**) An overview graph was plotted to reveal the relationships between the resistance mechanisms of ARGs and microbes. The left inner circle of the diagram represents the cumulative number of ARGs within a phylum that possess resistance mechanisms of this type. In contrast, the outer circle represents the relative proportion of ARGs within each phylum that belong to their respective resistance mechanism. The right inner circle displays the cumulative number of ARGs in a phylum across different resistance mechanisms. In contrast, the outer circle illustrates the relative proportion of ARGs within each resistance mechanism that belong to their respective phylum. n = 3 per vector. Microbes with black and red fonts indicate that they belong to the kingdoms of Bacteria and Eukaryotas, respectively.

After a comprehensive genome-wide analysis of microbiota, including bacteria, eukaryotes, archaea, and viruses in four different vectors, we next focused on the ARGs. The absolute abundances of ARGs in four vectors are shown in Figure 7A on the left. No significant difference was found between the microbial gene numbers. Nevertheless, the number of antibiotic resistance ontologies (AROs) was consistent with the microbial gene number results (Figure 7A right). It was significantly higher in PM and soil than in dung and fodder (*p* < 0.01) and was lowest in fodder (*p* < 0.01). The Venn diagram shows the number of standard and unique AROs in four vectors (Figure 7B). A total of 117 common AROs were found in the four vectors. The number of unique AROs in fodder was the highest, with 33, followed by dung, with 28. The number of AROs unique to PM and soil was similar, with 14 and 10, respectively.

It can be seen from Figure 6C that the relative abundance of the 20 most dominant ARGs in the dung and fodder varied considerably within the groups. The abundances of each sample in the soil and PM groups were close. Figure 7C,D show that the variety of ARGs in the fodder samples was less than in the other vectors. In terms of the components, the rifampicin (*rphB*), fluoroquinolone (*mexD*), and aminoglycosides (*aph3′-ia*) resistance genes were dominant ARGs detected in fodder. The other three vectors had more abundant ARGs, which were completely different from those in fodder, mainly tetracycline (*tetQ*, *tet40*, *tetL*, *tet32*, *tet36*, *tetW/N/W*), macrolides (*ermA*, *ermB*, *ermC*, *ermF*) and florfenicols (*floR*, *optrA*) resistance genes. To provide a more intuitive representation and comprehensive observation of the abundance proportions and overall distribution of ARGs within various samples, an overview circular plot was generated based on the abundances of the top 10 most dominant ARGs (Figure 7E). Consistent with the component analysis results, the *rphB*, *mexD* and *aph3′-ia* were detected only in the fodder samples, while the other ARGs could be found in PM, soil and dung samples.

To better understand the mechanisms by which microbiota develop resistance, a classification of resistance mechanisms associated with ARGs was conducted based on the CARD database. A resistance mechanism distribution chart was generated to elucidate the ARG mechanisms of microbiota (Figure 7F). The main resistance mechanisms in the three dominant bacteria were efflux, inactivation, and target alteration. They accounted for 30%, 30%, and 20% of Firmicutes, respectively. In Proteobacteria, efflux accounted for more than 60% of the resistance mechanisms, and inactivation and target alteration accounted for about 20% and 10%, respectively. Different from these two dominant bacteria, inactivation accounted for the highest proportion, more than 40% in Bacteroidetes. In dominant eukaryote of Ascomycota, efflux, inactivation, and target replacement are the main resistance mechanisms. It is disparate in Basidiomycota that target alteration and target protection are the main resistance mechanisms.

### 3.7. Featured ARGs in Different Vectors

**Figure 8:**
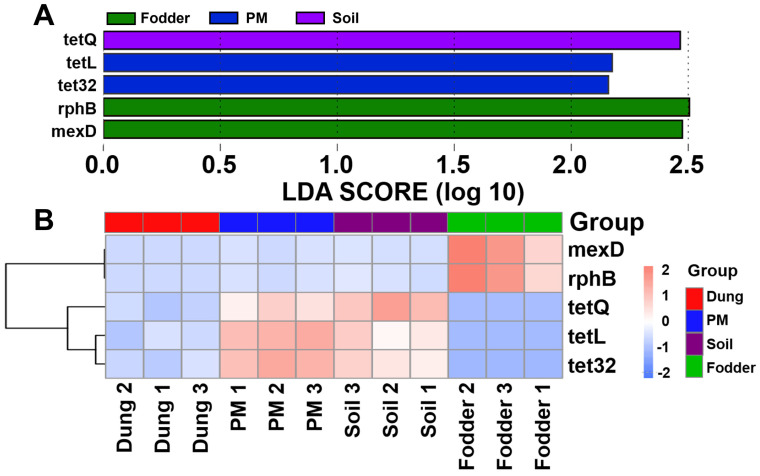
Screening of ARG biomarkers with significant differences among different vectors in the fattening swine house based on the LEfSe analysis. (**A**) The bar chart shows the LDA score of ARGs in PM, soil, and fodder. (**B**) Clustering heatmap of differential ARGs. Significant differences were defined as *p* < 0.05 and LDA score > 2.0. n = 3 per vector.

Five featured ARGs were analyzed. They are *tetQ* in soil, *tetL* and *tet32* in PM, *rphB,* and *mexD* in fodder, respectively (Figure 8A). No featured ARGs were screened in dung. The heatmap also uncovered that the ARGs of *mexD* and *rphB* were abundant only in fodder samples. The tetracycline ARGs of *tetQ*, *tetL,* and *tet32* were abundant in both PM and soil samples (Figure 8B). But it can still be seen that *tetQ* was more abundant in soil, while *tetL* and *tet32* were more abundant in PM. Additionally, there are no featured ARGs that are abundant only in dung.

### 3.8. ARGs and Microbiota Attribution

**Figure 9:**
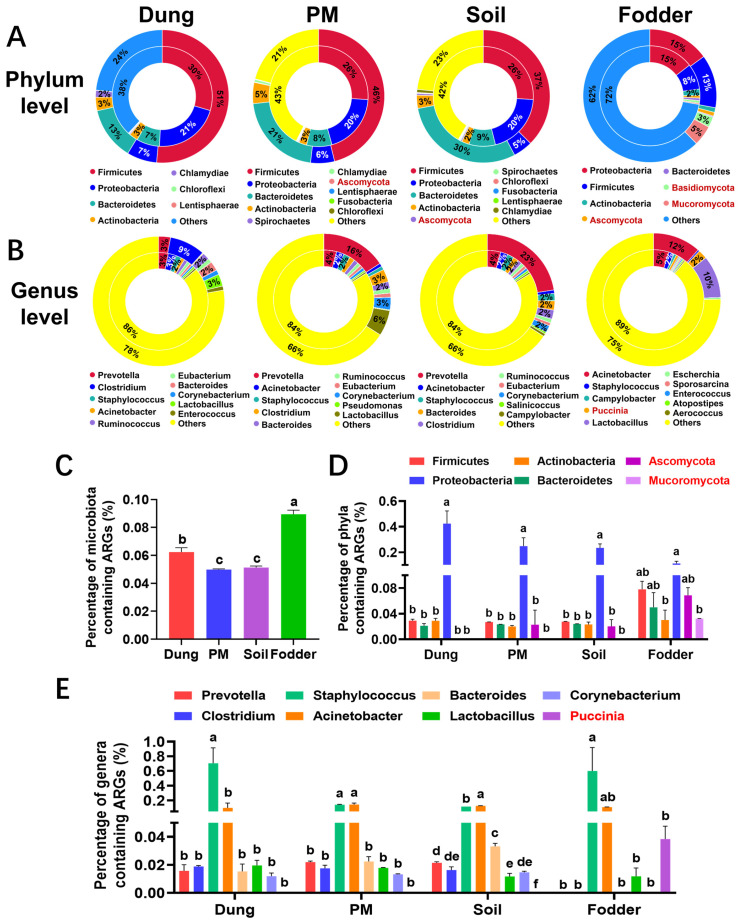
Identification circle diagram of the attribution of ARGs on phylum level (**A**) and genus level (**B**) among different vectors in the fattening swine house. The inner circle is the distribution of ARGs, and the outer circle is the distribution of genes in all samples in the group. (**C**) Percentage of microbiota containing ARGs among different vectors. (**D**) Percentage of 6 most dominant phyla containing ARGs among different vectors. (**E**) Percentage of 8 dominant genera containing ARGs among different vectors. n = 3 per vector. Different small letters represent significant differences between vectors (*p* < 0.05); the same letters show no significant differences (*p* > 0.05). Microbes with black and red fonts indicate that they belong to the kingdoms of bacteria and eukaryotas, respectively.

The outer circle of the microbiota attribution circle represents the species distribution of all genes, which is the same as the microbiota composition in Figure 3. The inner circle displays the species distribution of ARGs. The comparison of the inner and outer circles reflects which microbiota contain more ARGs. It can be seen that the Firmicutes accounted for 51%, 46%, 37%, 13%, and Proteobacteria accounted for 7%, 6%, 5%, 15% among the phyla detected in dung, PM, and soil, respectively (Figure 9A). However, Firmicutes only accounted for 30%, 26%, 26%, 8%. In comparison, Proteobacteria accounted for 21%, 20%, 20%, and 15% among the phyla carrying ARGs, indicating that Proteobacteria were more likely to contain ARGs than Firmicutes.

At the genus level, the dominant genus *Clostridium* in dung accounted for 9%, while it accounted for only 3% among the genera carrying ARGs (Figure 9B). The dominant genus *Prevotella* accounted for 16% and 23% in PM and soil, respectively, while it accounted for only 4% and 4% among the genera carrying ARGs. In the fodder, the dominant genera *Acinetobacter* and *Lactobacillus* accounted for 12% and 10%, respectively. In comparison, they accounted for only 5% and 0.41% among the genera carrying ARGs. *Staphylococcus* was not the dominant genus in the four vectors, accounting for only 0.06%, 0.88%, 1.76%, and 0.40%, respectively. However, its proportion in the genera containing ARGs was as high as 2.0%, 2.1%, 2.1% and 1.7%, respectively. These results indicated that *Staphylococcus* carried more ARGs than *Clostridium*, *Prevotella*, *Acinetobacter*, and *Lactobacillus* in the four vectors.

We then analyzed the proportion of microbiota carrying ARGs in different vectors (Figure 9C). It was found that the proportion of microbiota carrying ARGs in fodder was significantly higher than that in other vectors (*p* < 0.05), followed by dung, soil, and PM. In order to intuitively reflect which microbiota in different vectors are more likely to carry ARGs, we counted the percentage of six dominant phyla (Figure 9D) and eight dominant genera (Figure 9E) containing ARGs. The results showed that at the phylum level, Proteobacteria in dung, PM, and soil carried significantly more ARGs than other bacteria (*p* < 0.05), 9- to 20-fold more than other bacteria. There was little difference in the ability of microbiota in the fodder to carry ARGs. However, Proteobacteria still had the highest proportion of ARGs, 1.4–3.7 times higher than the others.

At the genus level, *Staphylococcus* and *Acinetobacter* carry the highest ARGs. In dung and fodder, the proportion of *Staphylococcus* carrying ARGs was significantly higher than that of other genera (*p* < 0.05). The genera of *Prevotella*, *Clostridium*, *Bacteroides*, and *Corynebacterium* had low content. They were not detected to carry ARGs in the fodder. In PM and soil, the proportion of *Acinetobacter* carrying ARGs was similar to that of *Staphylococcus* and significantly higher than that of other genera (*p* < 0.05).

### 3.9. Absolute Quantification of Major ARGs

**Figure 10:**
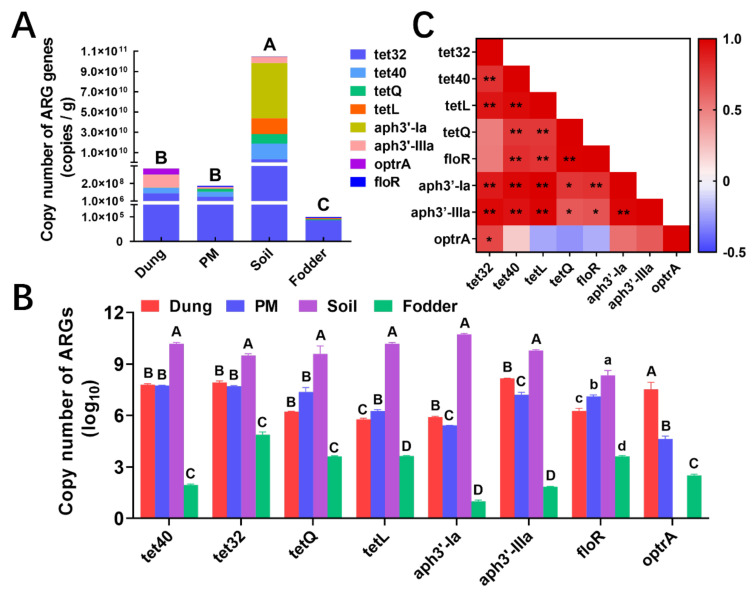
Absolute quantification of major ARGs in dung, PM, soil, and fodder in the fattening swine house. (**A**) Absolute abundance of ARGs in different environmental vectors. Different capital letters represent significant differences between vectors (*p* < 0.01); the same letters show no significant differences (*p* > 0.05). (**B**) Comparison of ARG copy number (based on log10) in different environmental vectors. Different capital letters represent significant differences between vectors (*p* < 0.01); different lowercase letters represent significant differences between vectors (*p* < 0.05); the same letters show no significant differences (*p* > 0.05). (**C**) Correlation analysis of ARG abundance in environmental vectors by Pearson. * *p* < 0.05, ** *p* < 0.01. n = 3 per vector.

As shown in Figure 10A, the total content of the eight ARGs tested was lowest in fodder, which was significantly lower than the other vectors (*p* < 0.05), and highest in soil, which was significantly higher than the other vectors (*p* < 0.05). The *aph3′-iiia*, *tet40*, *aph3′-ia*, and *tet32* were the dominant ARGs in dung, PM, soil and fodder with the highest abundance of (1.47 ± 0.03) × 10^8^, (5.62 ± 0.20) × 10^7^, (5.46 ± 0.74) × 10^10^ and (8.68 ± 3.27) × 10^4^ copies/g, respectively (Table S4). In terms of different vectors, the highest expression of each ARG was observed in soil, which was significantly higher than that in the other vectors (*p* < 0.05, Figure 10B), except for *optrA*, which was not detected in soil, and highest in dung. The lowest expression was observed in fodder, which was significantly lower than in the other vectors (*p* < 0.05). Different ARGs (Figure S2) detected high levels of *tet32* and *tet40* in dung and PM. High levels of *aph3′-ia* and *tet40* in soil but low in fodder were observed. In fodder, the content of *tet32* was the highest.

Pearson analyzed the correlation between different ARGs (Figure 10C), and it was shown that most ARGs had a significant positive correlation. The tetracycline (*tet32*, *tet40*) and aminoglycosides (*aph3′-ia*, *aph3′-iiia*) ARGs were significantly positively correlated. The *tet32* had a significant positive correlation with all ARGs (*p* < 0.05) except *tetQ* and *floR*. The *optrA* was only significantly positively correlated with *tet32* (*p* < 0.05) but not with other ARGs.

## 4. Discussion

Swine farms are one of the critical environmental pollution sources. More pathogens are often detected in swine houses than in ambient air [14], posing a safety risk. Therefore, attention should be paid to the environmental management of swine farms to reduce the risk of pathogenic bacteria transmission. To ensure the health of farmed animals, “Hygienic standards for feeds” (GB 13078-2017) [15] strictly limit the number of bacteria and fungi in fodder. The number of microbial genes in the fodder was minimal, as expected. Dung is the main solid waste, the primary source of environmental pollution, and an essential reservoir of resistant bacteria and ARGs in livestock farms [16]. More bacteria detected in soil than in dung indicates that soil is not only contaminated by dung but also serves as a shelter for many microorganisms. Few reports have focused on the number of microorganisms in airborne PM in swine houses. It is worth noting that airborne PM is rich in microbial genes, which was significantly higher than that in dung and close to that in soil, suggesting that it is a crucial risk factor for the transmission of pathogenic bacteria and affecting the respiratory health of livestock.

The soil and PM were the closest in microbial community structure. This can be explained by the fact that ground soil is one of the primary sources of airborne PM [17]. Their dominant genus, *Prevotella*, is a crucial player in the balance between health and disease [18], and they were also the differential and featured genus in this study. The microbial composition of the fodder was visually distinguished from the other three because it contained a relatively high abundance of fungi based on the differential and featured microbes. The eukaryotes of Mucoromycota [19], Basidiomycota [20], Ascomycota [21], and the genus of *Rhizophagus* [22] were the typical roots of plant-interacting fungi. It indicates that it comes from fodder raw materials such as corn and soybean. Therefore, the control of fungi in fodder ingredients is vital for the health of farm animals. The microbial composition in the dung was also clearly distinguished from the other three. Even within-group samples were highly variable, indicating that individual differences in fattening pigs were significant. We noted that archaeal of Euryarchaeota abundance in dung was 3–6 times higher than in PM and soil and up to 94 times higher than in fodder, which plays a previously underestimated role in human health and disease [23]. The dominant genus *Clostridium* was gut symbiont, which is closely related to bowel disease and health [24]. Besides bacteria, two featured archaeal species belonging to *Methanobrevibacter*, which is an extreme anaerobe that can be isolated in the gastrointestinal tract [25], were found in dung. Also, one viral species of uncultured_crAssphage was detected, suggesting that dung is a potential source of viral infection if not cleaned in swine houses.

In a study of 147 fecal samples collected from 62 swine farms, 20 cattle farms, and 19 poultry farms, swine farms were found to be the most contaminated with ARGs [26]. A total of 524 ARGs were detected in four vectors in the detected fattening house, which was a massive amount of pollution. At the time of sample collection in this experiment, antibiotics were still added to fodder as additives before the official implementation of the anti-resistance policy on 1 January 2020 in China. The number of microbial genes in the fodder was significantly lower than in the other vectors. However, the number of ARGs was not, indicating that the proportion of resistant microorganisms in the fodder was higher than that in the other vectors, which was verified in Figure 9C. The soil may be generally contaminated with ARGs [27], where the fodder raw materials, like corn and soybean, grow. The high use of antibiotic additives in fodder may also lead to the development of ARGs in the microorganisms therein. Intake of such fodder can significantly promote the production of ARGs in animal bodies. However, the number of AROs detected in the fodder was significantly lower than that of other vectors, probably due to the limited types of antibiotics added.

The ARGs in dung were significantly different from those in fodder but closer to those in PM and soil, indicating that fodder is only a tiny part of the source of ARGs in animals. The surrounding environment is a more critical factor. Among the top 20 abundant ARGs, tetracycline ARGs had the highest abundance, followed by aminoglycosides, rifampicin, and macrolides ARGs, which were attributed to the heavy use of these antibiotics in the experimental farm. The high detection rate and abundance of these ARGs were also detected in other swine farms, indicating that they were commonly used for disease control and growth promotion in swine farms before the ban [28,29]. These most prevalent ARGs are produced and spread rapidly, mainly through efflux, inactivation, and target alteration mechanisms. The floR gene, an efflux gene that can mediate florfenicol resistance, was identified in a fish-derived *Pasteurella piscicida* strain by Kim et al. in 1996 [30], only 6 years after florfenicol was first used in aquaculture in 1990. Worrisome is that these ARGs can spread not only vertically but also horizontally. The structure of ARGs on plasmids is unstable and can be transmitted between bacterial populations. They do not disappear even when the host bacteria die. However, they are released into the environment to be taken up by other bacteria and exert their resistance role again [31].

Soil is one of the primary sources of airborne PM, in which microorganisms and ARGs can attach to PM and enter the air to complete soil–air migration [32]. Where the abundance of ARGs in the soil is high, like swine farms, it will directly promote the migration of ARGs to the air. In turn, the PM deposition will also affect the types and contents of ARGs in soil. Among the vectors for pairwise comparison, airborne PM and soil shared the most mutual genes and the most similar ARG compositions, confirming the exchange of ARGs between them. Previous studies have suggested that dung from swine farms is the primary source of ARG pollution, followed by soil and, finally, air [14]. However, we found that ARO quantities in soil and PM are significantly higher than that in dung in this study, indicating that soil and PM have other sources of contamination besides dung. The air from outside is one of the primary sources of airborne PM in swine houses [16] and is considered an essential route of ARGs [33]. Other farms near the detected barn may have become an essential source of ARG contamination in the airborne PM, explaining the more significant number of AROs in the airborne PM than in the dung inside the fattening house. Visibly, PM has the characteristics of long-term suspension in the air and transmission over a long distance with the airflow. It has become an essential contributor to the rapid long-distance transmission of ARG, reminding us of its detriment as a rich reservoir and carrier of ARGs.

The microbiota carrying the most ARGs were Firmicutes and Proteobacteria; the former was the dominant phyla in each vector, but the latter was not. This indicates that Proteobacteria are more likely to carry ARGs, especially in dung, PM, and soil. We statistically show that the proportion of Proteobacteria carrying ARG is 9- to 20-fold higher than other bacteria (Figure 9D). This is a precious finding, suggesting that Proteobacteria is extremely sensitive to ARGs. We can then pay more attention to the resistance of Proteobacteria or take targeted measures to reduce resistance from Proteobacteria, which may be a breakthrough to inhibit the spread of ARGs. At the genus level, *Staphylococcus* and *Acinetobacter*, which belong to Firmicutes and Proteobacteria, should be emphasized. *Acinetobacter* is a Gram-negative bacteria mainly associated with acquired infections, including pneumonia, bacteremia, endocarditis, skin and soft tissue infections, urinary tract infections, and meningitis [34]. The *Staphylococci* are Gram-positive bacteria, which can be divided into *Staphylococcus aureus* and non-*Staphylococcus aureus*. The former is one of the leading causes of hospital-acquired infections [35]. Animal diseases infected by these two genera will be more challenging to control because they are more likely to carry ARGs. Other genera carrying more ARGs due to their high abundance should also receive more attention, such as *Prevotella* and *Clostridium*, which are potential opportunistic pathogens. The ability of fungi in fodder to carry ARGs should also not be underestimated; ARGs are also highly expressed in Ascomycota and *Puccinia*. Some bacteria are abundant in the vector but carry few ARGs, such as *Lactobacillus*. This is related to the low use of antibiotics to kill such bacteria, as they are beneficial or harmless.

Combining the results of metagene analysis and the use of antibiotics on the farm, we selected eight representative ARGs in the four vectors for absolute quantitative analysis. The abundance of detected ARGs was highest in soil (7.67 × 10^10^~1.39 × 10^11^) and lowest in fodder (5.48 × 10^4^~1.63 × 10^5^), consistent with the metagene results. However, in more studies, the abundance of ARGs in soil was lower than that in dung [14], which might be the primary source of ARGs in soil in those farms, while soil had other vital sources of ARGs in this study, which deserves further investigation. The tetracycline ARGs were the most abundant detected in all vectors, especially *tet40* and *tet32* in dung, indicating that they are the key ARGs leading to the resistance of animal enteric microorganisms. Other swine farms are also in the majority, with tetracycline-class ARGs reported [36,37]. Most of these detected ARGs, even in different classes and with different mechanisms, had significant positive correlations with each other, except for optrA, which was only associated with *tet32*. These results indicated that the main ARGs in these farms could promote the transmission of each other, and there is a risk of horizontal gene transfer of ARGs [13].

## 5. Conclusions

In conclusion, the abundance of microorganisms and ARGs in the soil and PM in the fattening swine house was the highest. The composition was also similar, both containing a high abundance of opportunistic pathogen *Prevotella* and tetracycline ARGs, so importance should be attached to the risks posed to animals and farmworkers. The lowest abundance of microorganisms and ARGs was found in the fodder, while it harbored the richest fungi. Among the dominant microorganisms in the detected vectors, Proteobacteria are the most likely to carry ARGs, with the primary resistance mechanism of efflux. These results may provide a new direction for effectively controlling the spread of antibiotic resistance and reducing the emergence of multi-drug-resistant pathogens.

## Data Availability

Raw metagenome sequencing data from the SRA database with accession number PRJNA1110558 are available.

## Supplementary Materials

The following supporting information can be downloaded at: https://www.mdpi.com/article/10.3390/toxics12120916/s1, Table S1: Sequences and parameters of gene primers used for quantitative real-time; Table S2: Linear regression equation of target ARGs.
