# Supplementary material for: Contamination Characteristics of Antibiotic Resistance Genes in Multi-Vector Environment in Typical Regional Fattening House
Source: Toxics. 2024 Dec 18;12(12):916. doi: 10.3390/toxics12120916 (PMC11728509; doi:10.3390/toxics12120916)
Supplement: Supplementary file 1 [file toxics-12-00916-s001.zip › toxics-3287665-supplementary.pdf]

Table S1. Sequences and parameters of gene primers used for quantitative real-time PCR.

Gene		Primer sequences (5'→3')	Annealing temperature (℃)
tet32	FW	CCATTACTTCGGACAACGGTAGA	59.5
	RV	CAATCTCTGTGAGGGCATTTAACA	
tet40	FW	GGCGCTCTTAGCAATCACAC	59.5
	RV	GGGCGATGAGCAACTGAGAA	
tetQ	FW	AGAATCTGCTGTTTGCCAGTG	54.5
	RV	CGGAGTGTCAATGATATTGCA	
tetL	FW	TCGTTAGCGTGCTGTCATTC	55
	RV	GTATCCCACCAATGTAGCCG	
aph3'-ia	FW	ATCGCGTATTTCGTCTCGCT	59
	RV	GGAGAAAACTCACCGAGGCA	
aph3'-iiia	FW	GCCGGTATAAAGGGACCACC	59
	RV	ACTCTTCCGAGCAAAGGACG	
floR	FW	CCAACTCACGTTGAGCCTCT	60
	RV	CATAAACGTCGCGAACCGTC	
optrA	FW	AAACACTTATGGGTGGTGTGG	60
	RV	CTGAAATGAGCCAAGAGCAG	

Table S2. Linear regression equation of target ARGs.

Gene	Linear equation	Regression coefficient (R^2)
tet32	Y=-3.503*X+45.09	0.9995
tet40	Y=-3.092*X+41.74	0.9996
tetQ	Y=-2.879*X+38.16	0.9957
tetL	Y=-3.265*X+38.81	0.9988
aph3'-ia	Y=-2.569*X+32.90	0.9936
aph3'-iiia	Y=-3.081*X+39.86	0.9991
floR	Y=-3.584*X+44.31	0.9962
optrA	Y=-3.296*X+40.06	0.9943

Table S3. The detailed functions and KEGG [1] pathways of 74 featured proteins.

No.	Group	Symbol	Name	Level 1	Level 2	Level 3
1	**Soil**	**K07133**	uncharacterized protein	Not Included in Pathway or Brite	Poorly characterized	Function unknown
2		**K21572**	starch-binding outer membrane protein, SusD/RagB family	Brite Hierarchies	Protein families: signaling and cellular processes	Transporters
3		**K03088**	rpoE; RNA polymerase sigma-70 factor, ECF subfamily	Brite Hierarchies	Protein families: genetic information processing	Transcription machinery
4		**K01190**	lacZ; beta-galactosidase	Metabolism	Carbohydrate metabolism	Galactose metabolism
					Lipid metabolism	Sphingolipid metabolism
					Glycan biosynthesis and metabolism	Other glycan degradation
5		**K18220**	tetM, tetO; ribosomal protection tetracycline resistance protein	Brite Hierarchies	Protein families: signaling and cellular processes	Antimicrobial resistance genes
6		**K05349**	bglX; beta-glucosidase	Metabolism	Carbohydrate metabolism	Starch and sucrose metabolism
					Metabolism of other amino acids	Cyanoamino acid metabolism
					Biosynthesis of other secondary metabolites	Degradation of flavonoids
						Biosynthesis of various plant secondary metabolites
7		**K03655**	recG; ATP-dependent DNA helicase RecG	Genetic Information Processing	Replication and repair	Homologous recombination
				Brite Hierarchies	Protein families: genetic information processing	DNA repair and recombination proteins
8		**K06921**	K06921; uncharacterized protein	Not Included in Pathway or Brite	Poorly characterized	Function unknown
9	**PM**	**K07498**	K07498; putative transposase	Not Included in Pathway or Brite	Unclassified: genetic information processing	Replication and repair
10	**Dung**	**K06147**	ABCB-BAC; ATP-binding cassette, subfamily B, bacterial	Brite Hierarchies	Protein families: signaling and cellular processes	Transporters
11		**K01990**	ABC-2.A; ABC-2 type transport system ATP-binding protein	Brite Hierarchies	Protein families: signaling and cellular processes	Transporters
12		**K02003**	ABC.CD.A; putative ABC transport system ATP-binding protein	Brite Hierarchies	Protein families: signaling and cellular processes	Transporters
13		**K01520**	dut, DUT; dUTP diphosphatase	Metabolism	Nucleotide metabolism	Pyrimidine metabolism
					Xenobiotics biodegradation and metabolism	Drug metabolism - other enzymes
14		**K02337**	dnaE; DNA polymerase III	Genetic Information	Replication and repair	DNA replication

			subunit alpha	Processing		Mismatch repair
						Homologous recombination
				Brite Hierarchies	Protein families: genetic information processing	DNA replication proteins
						DNA repair and recombination proteins
15		**K07491**	rayT; REP-associated tyrosine transposase	Not Included in Pathway or Brite	Unclassified: genetic information processing	Replication and repair
16		**K03701**	uvrA; excinuclease ABC subunit A	Genetic Information Processing	Replication and repair	Nucleotide excision repair
				Brite Hierarchies	Protein families: genetic information processing	DNA repair and recombination proteins
17		**K05573**	ndhB; NAD(P)H-quinone oxidoreductase subunit 2	Metabolism	Energy metabolism	Oxidative phosphorylation
18		**K05572**	ndhA; NAD(P)H-quinone oxidoreductase subunit 1	Metabolism	Energy metabolism	Oxidative phosphorylation
19		**K14175**	NES1; (3S,6E)-nerolidol synthase	Metabolism	Metabolism of terpenoids and polyketides	Sesquiterpenoid and triterpenoid biosynthesis
20		**K01601**	rbcL, cbbL; ribulose-bisphosphate carboxylase large chain	Metabolism	Carbohydrate metabolism	Glyoxylate and dicarboxylate metabolism
					Energy metabolism	Carbon fixation in photosynthetic organisms
21		**K02689**	psaA; photosystem I P700 chlorophyll a apoprotein A1	Metabolism	Energy metabolism	Photosynthesis
				Brite Hierarchies	Protein families: metabolism	Photosynthesis proteins
22		**K05579**	ndhH; NAD(P)H-quinone oxidoreductase subunit H	metabolism	Energy metabolism	Oxidative phosphorylation
23	**Fodder**	**K02703**	psbA; photosystem II P680 reaction center D1 protein	Metabolism	Energy metabolism	Photosynthesis
				Brite Hierarchies	Protein families: metabolism	Photosynthesis proteins
24		**K02967**	RP-S2, MRPS2, rpsB; small subunit ribosomal protein S2	Genetic Information Processing	Translation	Ribosome
				Brite Hierarchies	Protein families: genetic information processing	Ribosome
25		**K02111**	ATPF1A, atpA; F-type H+/Na+-transporting ATPase subunit alpha	Metabolism	Energy metabolism	Oxidative phosphorylation
						Photosynthesis
				Brite Hierarchies	Protein families: metabolism	Photosynthesis proteins
26		**K02878**	RP-L16, MRPL16, rplP; large subunit ribosomal protein L16	Genetic Information Processing	Translation	Ribosome
				Brite Hierarchies	Protein families: genetic information processing	Ribosome
27		**K02690**	psaB; photosystem I P700 chlorophyll a apoprotein A2	metabolism	Energy metabolism	Photosynthesis
				Brite Hierarchies	Protein families: metabolism	Photosynthesis proteins
28		**K05574**	ndhC; NAD(P)H-quinone oxidoreductase subunit 3	metabolism	Energy metabolism	Oxidative phosphorylation
29		**K05578**	ndhG; NAD(P)H-quinone	metabolism	Energy metabolism	Oxidative phosphorylation

			oxidoreductase subunit 6			
30		**K05582**	ndhK; NAD(P)H-quinone oxidoreductase subunit K	Metabolism	Energy metabolism	Oxidative phosphorylation
31		**K02704**	psbB; photosystem II CP47 chlorophyll apoprotein	Metabolism	Energy metabolism	Photosynthesis
				Brite Hierarchies	Protein families: metabolism	Photosynthesis proteins
32		**K05577**	ndhF; NAD(P)H-quinone oxidoreductase subunit 5	Metabolism	Energy metabolism	Oxidative phosphorylation
33		**K05580**	ndhI; NAD(P)H-quinone oxidoreductase subunit I	Metabolism	Energy metabolism	Oxidative phosphorylation
34		**K05581**	ndhJ; NAD(P)H-quinone oxidoreductase subunit J	Metabolism	Energy metabolism	Oxidative phosphorylation
35		**K02635**	petB; cytochrome b6	Metabolism	Energy metabolism	Photosynthesis
				Brite Hierarchies	Protein families: metabolism	Photosynthesis proteins
36		**K02112**	ATPF1B, atpD; F-type H+/Na+-transporting ATPase subunit beta	Metabolism	Energy metabolism	Oxidative phosphorylation
					Energy metabolism	Photosynthesis
				Brite Hierarchies	Protein families: metabolism	Photosynthesis proteins
37		**K05575**	ndhD; NAD(P)H-quinone oxidoreductase subunit 4	Metabolism	Energy metabolism	Oxidative phosphorylation
38		**K02634**	petA; apocytochrome f	Metabolism	Energy metabolism	Photosynthesis
				Brite Hierarchies	Protein families: metabolism	Photosynthesis proteins
39		**K02126**	ATPeF0A, MTATP6, ATP6; F-type H+-transporting ATPase subunit a	Metabolism	Energy metabolism	Oxidative phosphorylation
				Organismal Systems	Environmental adaptation	Thermogenesis
				Human Diseases	Cancer: overview	Chemical carcinogenesis - reactive oxygen species
					Neurodegenerative disease	Alzheimer disease
						Parkinson disease
						Amyotrophic lateral sclerosis
						Huntington disease
						Prion disease
						Pathways of neurodegeneration - multiple diseases
					Cardiovascular disease	Diabetic cardiomyopathy
				Brite Hierarchies	Protein families: genetic information processing	Mitochondrial biogenesis
40		**K17604**	ZSWIM3; zinc finger SWIM domain-containing protein 3	Brite Hierarchies	Protein families: metabolism	Protein phosphatases and associated proteins
41		**K15255**	PIF1; ATP-dependent DNA helicase PIF1	Brite Hierarchies	Protein families: genetic information processing	DNA replication proteins
					Protein families: genetic information processing	Mitochondrial biogenesis
42		**K02706**	psbD; photosystem II P680	Metabolism	Energy metabolism	Photosynthesis

			reaction center D2 protein	Brite Hierarchies	Protein families: metabolism	Photosynthesis proteins
43		**K03935**	NDUFS2; NADH dehydrogenase (ubiquinone) Fe-S protein 2	Metabolism	Energy metabolism	Oxidative phosphorylation
				Organismal Systems	Nervous system	Retrograde endocannabinoid signaling
					Environmental adaptation	Thermogenesis
				Human Diseases	Cancer: overview	Chemical carcinogenesis - reactive oxygen species
					Neurodegenerative disease	Alzheimer disease
						Parkinson disease
						Amyotrophic lateral sclerosis
						Huntington disease
						Prion disease
						Pathways of neurodegeneration - multiple diseases
					Endocrine and metabolic disease	Non-alcoholic fatty liver disease
					Cardiovascular disease	Diabetic cardiomyopathy
44		**K02705**	psbC; photosystem II CP43 chlorophyll apoprotein	Metabolism	Energy metabolism	Photosynthesis
				Brite Hierarchies	Protein families: metabolism	Photosynthesis proteins
45		**K02637**	petD; cytochrome b6-f complex subunit 4	Metabolism	Energy metabolism	Photosynthesis
				Brite Hierarchies	Protein families: metabolism	Photosynthesis proteins
46		**K03883**	ND5; NADH-ubiquinone oxidoreductase chain 5	Metabolism	Energy metabolism	Oxidative phosphorylation
				Organismal Systems	Nervous system	Retrograde endocannabinoid signaling
					Environmental adaptation	Thermogenesis
				Human Diseases	Cancer: overview	Chemical carcinogenesis - reactive oxygen species
					Neurodegenerative disease	Alzheimer disease
						Parkinson disease
						Amyotrophic lateral sclerosis
						Huntington disease
						Prion disease
						Pathways of neurodegeneration - multiple diseases
					Cardiovascular disease	Diabetic cardiomyopathy
				Brite Hierarchies	Protein families: genetic information processing	Mitochondrial biogenesis
47		**K05576**	ndhE; NAD(P)H-quinone oxidoreductase subunit 4L	Metabolism	Energy metabolism	Oxidative phosphorylation
48		**K02709**	psbH; photosystem II PsbH protein	Metabolism	Energy metabolism	Photosynthesis
				Brite Hierarchies	Protein families: metabolism	Photosynthesis proteins
49		**K02110**	ATPF0C, atpE; F-type H+-	Metabolism	Energy metabolism	Oxidative phosphorylation

			transporting ATPase subunit c		Energy metabolism	Photosynthesis
				Brite Hierarchies	Protein families: metabolism	Photosynthesis proteins
50		K02261	COX2; cytochrome c oxidase subunit 2	Metabolism	Energy metabolism	Oxidative phosphorylation
				Organismal Systems	Circulatory system	Cardiac muscle contraction
					Environmental adaptation	Thermogenesis
				Human Diseases	Cancer: overview	Chemical carcinogenesis - reactive oxygen species
					Neurodegenerative disease	Parkinson disease
						Amyotrophic lateral sclerosis
						Huntington disease
						Prion disease
						Pathways of neurodegeneration - multiple diseases
					Endocrine and metabolic disease	Non-alcoholic fatty liver disease
					Cardiovascular disease	Diabetic cardiomyopathy
				Brite Hierarchies	Protein families: genetic information processing	Mitochondrial biogenesis
51		K03881	ND4; NADH-ubiquinone oxidoreductase chain 4	Metabolism	Energy metabolism	Oxidative phosphorylation
				Organismal Systems	Nervous system	Retrograde endocannabinoid signaling
					Environmental adaptation	Thermogenesis
				Human Diseases	Cancer: overview	Chemical carcinogenesis - reactive oxygen species
					Neurodegenerative disease	Alzheimer disease
						Parkinson disease
						Amyotrophic lateral sclerosis
						Huntington disease
						Prion disease
						Pathways of neurodegeneration - multiple diseases
					Cardiovascular disease	Diabetic cardiomyopathy
				Brite Hierarchies	Protein families: genetic information processing	Mitochondrial biogenesis
52		K02132	ATPeF1A, ATP5A1, ATP1; F-type H+-transporting ATPase subunit alpha	Metabolism	Energy metabolism	Oxidative phosphorylation
				Organismal Systems	Environmental adaptation	Thermogenesis
				Human Diseases	Cancer: overview	Chemical carcinogenesis - reactive oxygen species
					Neurodegenerative disease	Alzheimer disease
						Parkinson disease
						Amyotrophic lateral sclerosis
						Huntington disease

						Prion disease
						Pathways of neurodegeneration - multiple diseases
					Cardiovascular disease	Diabetic cardiomyopathy
				Brite Hierarchies	Protein families: genetic information processing	Mitochondrial biogenesis
53		**K02691**	psaC; photosystem I subunit VII	Metabolism	Energy metabolism	Photosynthesis
				Brite Hierarchies	Protein families: metabolism	Photosynthesis proteins
54		**K02712**	psbK; photosystem II PsbK protein	Metabolism	Energy metabolism	Photosynthesis
				Brite Hierarchies	Protein families: metabolism	Photosynthesis proteins
55		**K02518**	infA; translation initiation factor IF-1	Brite Hierarchies	Protein families: genetic information processing	Translation factors
56		**K02256**	COX1; cytochrome c oxidase subunit 1	Metabolism	Energy metabolism	Oxidative phosphorylation
				Organismal Systems	Circulatory system	Cardiac muscle contraction
					Environmental adaptation	Thermogenesis
				Human Diseases	Cancer: overview	Chemical carcinogenesis - reactive oxygen species
					Neurodegenerative disease	Parkinson disease
						Amyotrophic lateral sclerosis
						Huntington disease
						Prion disease
						Pathways of neurodegeneration - multiple diseases
					Endocrine and metabolic disease	Non-alcoholic fatty liver disease
					Cardiovascular disease	Diabetic cardiomyopathy
				Brite Hierarchies	Protein families: genetic information processing	Mitochondrial biogenesis
57		**K02262**	COX3; cytochrome c oxidase subunit 3	Metabolism	Energy metabolism	Oxidative phosphorylation
				Organismal Systems	Circulatory system	Cardiac muscle contraction
					Environmental adaptation	Thermogenesis
				Human Diseases	Cancer: overview	Chemical carcinogenesis - reactive oxygen species
					Neurodegenerative disease	Parkinson disease
						Amyotrophic lateral sclerosis
						Huntington disease
						Prion disease
						Pathways of neurodegeneration - multiple diseases
					Endocrine and metabolic disease	Non-alcoholic fatty liver disease
					Cardiovascular disease	Diabetic cardiomyopathy

				Brite Hierarchies	Protein families: genetic information processing	Mitochondrial biogenesis
58		**K02707**	psbE; photosystem II cytochrome b559 subunit alpha	Metabolism	Energy metabolism	Photosynthesis
				Brite Hierarchies	Protein families: metabolism	Photosynthesis proteins
59		**K03882**	ND4L; NADH-ubiquinone oxidoreductase chain 4L	Metabolism	Energy metabolism	Oxidative phosphorylation
				Organismal Systems	Nervous system	Retrograde endocannabinoid signaling
					Environmental adaptation	Thermogenesis
				Human Diseases	Cancer: overview	Chemical carcinogenesis - reactive oxygen species
					Neurodegenerative disease	Alzheimer disease
						Parkinson disease
						Amyotrophic lateral sclerosis
						Huntington disease
						Prion disease
						Pathways of neurodegeneration - multiple diseases
					Cardiovascular disease	Diabetic cardiomyopathy
				Brite Hierarchies	Protein families: genetic information processing	Mitochondrial biogenesis
60		**K00430**	E1.11.1.7; peroxidase	Metabolism	Biosynthesis of other secondary metabolites	Phenylpropanoid biosynthesis
61		**K03878**	ND1; NADH-ubiquinone oxidoreductase chain 1	Metabolism	Energy metabolism	Oxidative phosphorylation
				Organismal Systems	Nervous system	Retrograde endocannabinoid signaling
					Environmental adaptation	Thermogenesis
				Human Diseases	Cancer: overview	Chemical carcinogenesis - reactive oxygen species
					Neurodegenerative disease	Alzheimer disease
						Parkinson disease
						Amyotrophic lateral sclerosis
						Huntington disease
						Prion disease
						Pathways of neurodegeneration - multiple diseases
					Cardiovascular disease	Diabetic cardiomyopathy
				Brite Hierarchies	Protein families: genetic information processing	Mitochondrial biogenesis
62		**K05658**	ABCB1, CD243; ATP-binding cassette, subfamily B (MDR/TAP), member 1	Environmental Information Processing	Membrane transport	ABC transporters
				Organismal Systems	Digestive system	Bile secretion
				Human Diseases	Cancer: overview	MicroRNAs in cancer
					Cancer: specific types	Gastric cancer

				Brite Hierarchies	Protein families: signaling and cellular processes	Transporters
						Exosome
						CD molecules
63		**K04733**	IRAK4; interleukin-1 receptor-associated kinase 4	Environmental Information Processing	Signal transduction	MAPK signaling pathway
						NF-kappa B signaling pathway
				Organismal Systems	Immune system	Toll-like receptor signaling pathway
						Toll and Imd signaling pathway
						NOD-like receptor signaling pathway
					Nervous system	Neurotrophin signaling pathway
				Human Diseases	Infectious disease: viral	Human immunodeficiency virus 1 infection
						Hepatitis B
						Coronavirus disease - COVID-19
						Influenza A
						Measles
						Herpes simplex virus 1 infection
						Epstein-Barr virus infection
					Infectious disease: bacterial	Pathogenic Escherichia coli infection
						Salmonella infection
						Yersinia infection
						Pertussis
						Tuberculosis
					Infectious disease: parasitic	Toxoplasmosis
						Leishmaniasis
						Chagas disease
					Cardiovascular disease	Lipid and atherosclerosis
					Endocrine and metabolic disease	Alcoholic liver disease
				Brite Hierarchies	Protein families: metabolism	Protein kinases
64		**K03880**	ND3; NADH-ubiquinone oxidoreductase chain 3	Metabolism	Energy metabolism	Oxidative phosphorylation
				Organismal Systems	Nervous system	Retrograde endocannabinoid signaling
					Environmental adaptation	Thermogenesis
				Human Diseases	Cancer: overview	Chemical carcinogenesis - reactive oxygen species
					Neurodegenerative disease	Alzheimer disease
						Parkinson disease
						Amyotrophic lateral sclerosis
						Huntington disease

						Prion disease
						Pathways of neurodegeneration - multiple diseases
					Cardiovascular disease	Diabetic cardiomyopathy
				Brite Hierarchies	Protein families: genetic information processing	Mitochondrial biogenesis
65		**K03879**	ND2; NADH-ubiquinone oxidoreductase chain 2	Metabolism	Energy metabolism	Oxidative phosphorylation
				Organismal Systems	Nervous system	Retrograde endocannabinoid signaling
					Environmental adaptation	Thermogenesis
				Human Diseases	Cancer: overview	Chemical carcinogenesis - reactive oxygen species
					Neurodegenerative disease	Alzheimer disease
						Parkinson disease
						Amyotrophic lateral sclerosis
						Huntington disease
						Prion disease
						Pathways of neurodegeneration - multiple diseases
					Cardiovascular disease	Diabetic cardiomyopathy
				Brite Hierarchies	Protein families: genetic information processing	Mitochondrial biogenesis
66		**K00337**	nuoH; NADH-quinone oxidoreductase subunit H	Metabolism	Energy metabolism	Oxidative phosphorylation
67		**K03884**	ND6; NADH-ubiquinone oxidoreductase chain 6	Metabolism	Energy metabolism	Oxidative phosphorylation
				Organismal Systems	Nervous system	Retrograde endocannabinoid signaling
					Environmental adaptation	Thermogenesis
				Human Diseases	Cancer: overview	Chemical carcinogenesis - reactive oxygen species
					Neurodegenerative disease	Alzheimer disease
						Parkinson disease
						Amyotrophic lateral sclerosis
						Huntington disease
						Prion disease
						Pathways of neurodegeneration - multiple diseases
					Cardiovascular disease	Diabetic cardiomyopathy
				Brite Hierarchies	Protein families: genetic information processing	Mitochondrial biogenesis
68		**K02954**	RP-S14, MRPS14, rpsN; small subunit ribosomal protein S14	Genetic Information Processing	Translation	Ribosome
				Brite Hierarchies	Protein families: genetic information processing	Ribosome

69		**K02887**	RP-L20, MRPL20, rplT; large subunit ribosomal protein L20	Genetic Information Processing	Translation	Ribosome
				Brite Hierarchies	Protein families: genetic information processing	Ribosome
70		**K02913**	RP-L33, MRPL33, rpmG; large subunit ribosomal protein L33	Genetic Information Processing	Translation	Ribosome
				Brite Hierarchies	Protein families: genetic information processing	Ribosome
71		**K00342**	nuoM; NADH-quinone oxidoreductase subunit M	Metabolism	Energy metabolism	Oxidative phosphorylation
72		**K13412**	CPK; calcium-dependent protein kinase	Organismal Systems	Environmental adaptation	Plant-pathogen interaction
				Brite Hierarchies	Protein families: metabolism	Protein kinases
73		**K02952**	RP-S13, rpsM; small subunit ribosomal protein S13	Genetic Information Processing	Translation	Ribosome
				Brite Hierarchies	Protein families: genetic information processing	Ribosome
74		**K14638**	SLC15A3_4, PHT, NPF8; solute carrier family 15	Brite Hierarchies	Protein families: signaling and cellular processes	Transporters

[1] The information was searched from the Kyoto Encyclopedia of Genes and Genomes (KEGG) database (http://www.kegg.jp/kegg/)

Table S4. Copy number of representative ARGs in four media in a fattening swine farm (copies/g).

Genes	Dung	PM	Soil	Fodder
**Tetracycline**				
tet32	$(8.82 \pm 1.65) \times 10^{7ab}$	$(5.13 \pm 0.36) \times 10^{7ab}$	$(3.31 \pm 0.85) \times 10^{9b}$	$(8.68 \pm 3.27) \times 10^{4a}$
tet40	$(6.36 \pm 0.86) \times 10^{7bc}$	$(5.62 \pm 0.20) \times 10^{7a}$	$(1.57 \pm 0.25) \times 10^{10b}$	$(9.02 \pm 1.25) \times 10^{b}$
tetQ	$(1.70 \pm 0.09) \times 10^{6c}$	$(3.41 \pm 2.08) \times 10^{7abc}$	$(9.17 \pm 6.67) \times 10^{9b}$	$(4.08 \pm 0.38) \times 10^{3b}$
tetL	$(6.19 \pm 0.81) \times 10^{5c}$	$(1.88 \pm 0.39) \times 10^{6c}$	$(1.56 \pm 0.23) \times 10^{10b}$	$(4.37 \pm 0.32) \times 10^{3b}$
**Aminoglycosides**				
aph3'-ia	$(8.27 \pm 0.73) \times 10^{5c}$	$(2.66 \pm 0.03) \times 10^{5c}$	$(5.46 \pm 0.74) \times 10^{10a}$	$(1.01 \pm 0.16) \times 10^{b}$
aph3'-iiia	$(1.47 \pm 0.03) \times 10^{8a}$	$(1.77 \pm 0.47) \times 10^{7bc}$	$(6.23 \pm 0.67) \times 10^{9b}$	$(7.03 \pm 0.45) \times 10^{b}$
**Florfenicols**				
floR	$(2.08 \pm 0.75) \times 10^{6c}$	$(1.34 \pm 0.27) \times 10^{7c}$	$(3.43 \pm 2.23) \times 10^{8b}$	$(4.17 \pm 0.47) \times 10^{3b}$
optrA	$(6.56 \pm 4.21) \times 10^{7bc}$	$(5.02 \pm 2.06) \times 10^{4c}$	-	$(3.32 \pm 0.59) \times 10^{2b}$

Each value represents the mean ± SEM of the group (n = 3).

Different small letters represent significant difference between media ($P < 0.05$); the same letters show no significant differences ($P > 0.05$).

"-" means no detected.

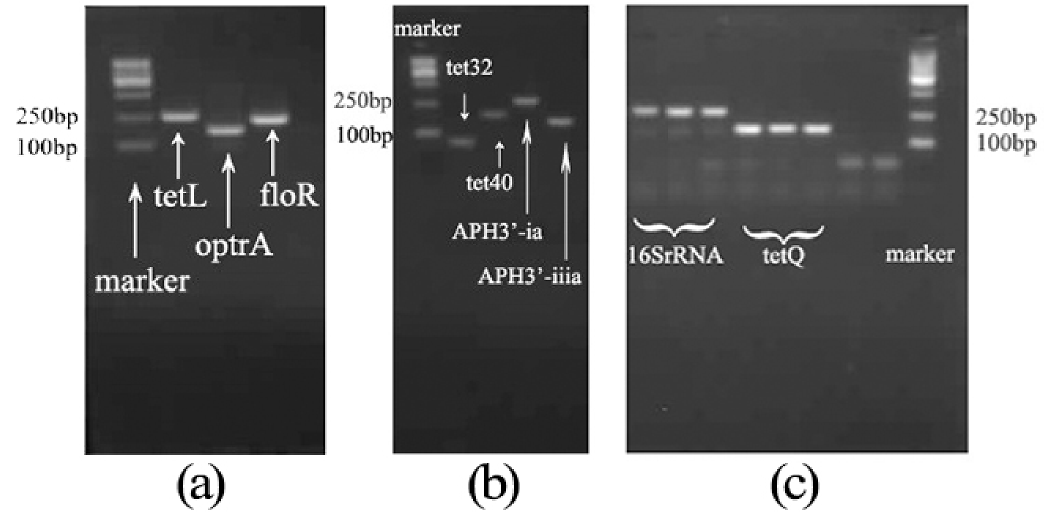

Figure S1. The purity and specificity of ARGs were tested by PCR-Agarose gel electrophoresis.

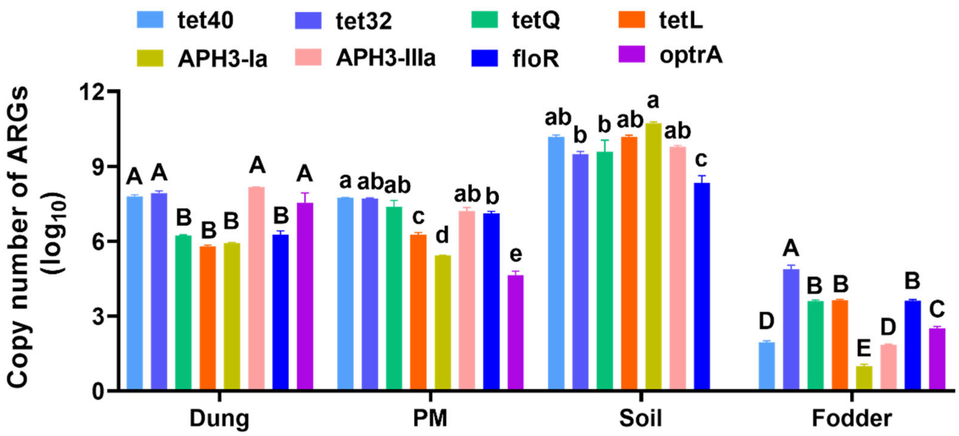

Figure S2. The copy number of each resistance gene in different media was compared. Comparison of copy numbers of different resistance genes in each medium. Different capital letters represent significant difference between media (P < 0.01); different lowercase letters represent significant difference between media (P < 0.05); the same letters show no significant differences (P > 0.05).
